# TPD52L2 Is a Prognostic Biomarker and Correlated With Immune Infiltration in Lung Adenocarcinoma

**DOI:** 10.3389/fphar.2021.728420

**Published:** 2021-10-19

**Authors:** Anyuan Zhong, Ting Chen, Tong Zhou, Zengli Zhang, Minhua Shi

**Affiliations:** Department of Pulmonary and Critical Care Medicine, The Second Affiliated Hospital of Soochow University, Suzhou, China

**Keywords:** TPD52L2, biomarker, lung adenocarcinoma, prognostic, immune infiltration, immunosuppressive

## Abstract

Tumor protein D52-like 2 (TPD52L2) belongs to the members of the TPD52 family. TPD52L2 was reported to regulate proliferation and apoptosis in cancer cells. However, its role in lung adenocarcinoma (LUAD) was uncertain. We evaluated the expression, methylation, copy number alteration, and prognostic significance of TPD52L2 using RNA-seq data from The Cancer Genome Atlas (TCGA). Enrichment analysis of TPD52L2 was conducted using the R package “clusterProfiler.” We further assessed the association between TPD52L2 and immune cell infiltration level, immunosuppressive genes, and tumor mutational burden (TMB). The difference of gene mutant frequency in high- and low-TPD52L2 groups was also analyzed. The results showed that TPD52L2 was over-expressed and predicted worse survival status in LUAD. We also found that TPD52L2 expression was positively associated with the infiltration levels of immunosuppressive cells, such as regulatory T cells (Tregs) and tumor-associated macrophages (TAMs), and negatively correlated with immune killer cells, such as CD8^+^ T and NK cells in pan-cancer, including LUAD. In addition, TPD52L2 expression was associated with immunosuppressive genes and TMB. High expression of TPD52L2 was with more mutant frequency of TP53. In summary, our results show that TPD52L2 is an oncogene and a potential prognostic biomarker in LUAD. High TPD52L2 expression is a possible indicator of immune infiltration and associated with tumor immunosuppressive status in LUAD.

## Introduction

Lung cancer is one of the most common malignant tumors, and more than 80% of them are non-small-cell lung cancer (NSCLC). Lung adenocarcinoma (LUAD) is the most frequent subtype of NSCLC worldwide, and the morbidity of LUAD has surpassed lung squamous carcinoma in recent years (Ferlay et al., 2018; Cao et al., 2020). Although target therapy and immunotherapy have improved the prognosis of LUAD patients, the five-year survival is still at a low level (Ferlay et al., 2019; Jurisic et al., 2020). Therefore, it is vital to indicate how to develop further effective therapeutic strategies to improve the survival of LUAD patients.

The tumor microenvironment (TME) refers to the environment where tumor cells originate and develop and is one of the main causes of malignant cancer occurrence and progression (Gajewski et al., 2013). Inflammatory cells make up a significant proportion of the overall tumor mass, and among them, macrophages, called tumor-associated macrophages (TAMs), which are particularly abundant in the TME, could alleviate tumor immunity and promote tumor progression (Li et al., 2019). In recent years, cancer immunotherapy has become a prominent cancer treatment, especially immune checkpoint inhibitors (Barbee et al., 2015). However, most patients of LUAD are still not sensitive to immune checkpoint inhibitors (Wang et al., 2020). Recent studies have shown that the remodeling of TME plays an important role in the development of LUAD, weakening the response of LUAD patients to immunotherapy (Jiang et al., 2021). Thus, the identification of essential genes that could affect the TME is urgently needed.

The tumor protein D52 (TPD52) family reportedly plays an important role in the proliferation and metastasis of various cancer cells (Sims et al., 2007; Ummanni et al., 2008; Abe et al., 2021). TPD52 family proteins are also considered novel candidate target proteins since these proteins are expressed in many types of cancers (Byrne et al., 2014). TPD52L2, a member of the TPD52-like protein family, is highly expressed and linked to poor prognosis in several types of cancers (Cheung et al., 2008; Ren et al., 2017; Qiang et al., 2018). In addition, TPD52L2 was also reported to regulate proliferation, apoptosis, and vehicle trafficking in a variety of tumors, including glioma (Wang et al., 2014), pancreatic adenocarcinoma (Chen et al., 2017), prostate cancer (Ren et al., 2017; Chi et al., 2018), and glioblastoma (Qiang et al., 2018). For example, the evaluated expression of TPD52L2 could accelerate the proliferation and invasiveness of glioma cells by regulating WNT signaling (Qiang et al., 2018), which provides a theoretical basis for the application of new medicine in tumor targeted therapy. However, the role of TPD52L2 in LUAD remains unclear, especially its correlation with the TME.

In our study, we comprehensively analyzed the role of TPD52L2 using pan-cancer data from TCGA database in 33 cancers, including expression, prognostic values, DNA methylation, copy number alteration (CNA), and mutation status of TPD52L2. The correlation between TPD52L2 expression and the infiltration level of immune cells, tumor mutational burden (TMB), and immunosuppressive genes was further evaluated in LUAD. This study revealed the potential role of TPD52L2 in tumor immunology and its prognostic value, which will propose a new target for tumor therapy.

## Materials and Methods

### Data Source

The RNA-seq data and corresponding clinical data of The Cancer Genome Atlas (TCGA), Genotype-Tissue Expression (GTEx), and Cancer Cell Line Encyclopedia (CCLE) were downloaded from the UCSC XENA website (https://xenabrowser.net/datapages/). The gene mutation data were obtained from the UCSC XENA website. The methylation and copy number of TPD52L2 were downloaded from the cBioPortal database (https://www.cbioportal.org/). The sample sizes from TCGA and GTEx databases are supplied in Supplementary Table S1.

### Prognostic Analysis

Univariate Cox regression (uniCox) and Kaplan–Meier analyses were conducted to explore the influence of TPD52L2 on the survival of patients with pan-cancer using R packages “survminer” and “survival.” Overall survival (OS), disease-specific survival (DSS), disease-free interval (DFI), and progression-free interval (PFI) were evaluated.

### Correlation Analysis and Enrichment Analysis

The correlation between TPD52L2 expression and all protein-coding mRNAs was analyzed in LUAD from TCGA cohort. The mRNAs correlated with TPD52L2 (Pearson’s correlation coefficient, *p* < 0.05) were ranked and subjected to gene set enrichment analysis (GSEA) using the R package “clusterProfiler.”

### Correlation Between TPD52L2 Expression and Immune Cell Infiltration

Two methods were used to assess the correlation between TPD52L2 expression and the infiltration level of immune cells. For the first method, we obtained the infiltration level of immune cells from a published work, which scores the infiltration level of 26 immune cells using “CIBERSOFT” (Thorsson et al., 2018). For the second method, we downloaded the infiltration data of 24 immune cells from the ImmuCellAI database (http://bioinfo.life.hust.edu.cn/ImmuCellAI#!/).

### Correlation Between TPD52L2 Expression and Tumor Mutational Burden (TMB) and Immunosuppressive Genes

TMB scores were determined for all samples based on somatic mutation data from TCGA and the correlation between TPD52L2 expression and TMB analyzed using Spearman’s rank correlation coefficient. In addition, we analyzed the correlation between TPD52L2 expression and immunosuppressive genes. In addition, the gene mutation data of LUAD were obtained from the UCSC XENA website and analyzed using the R package “maftools.”

## Results

### Pan-Cancer Expression of TPD52L2

**FIGURE 1:**
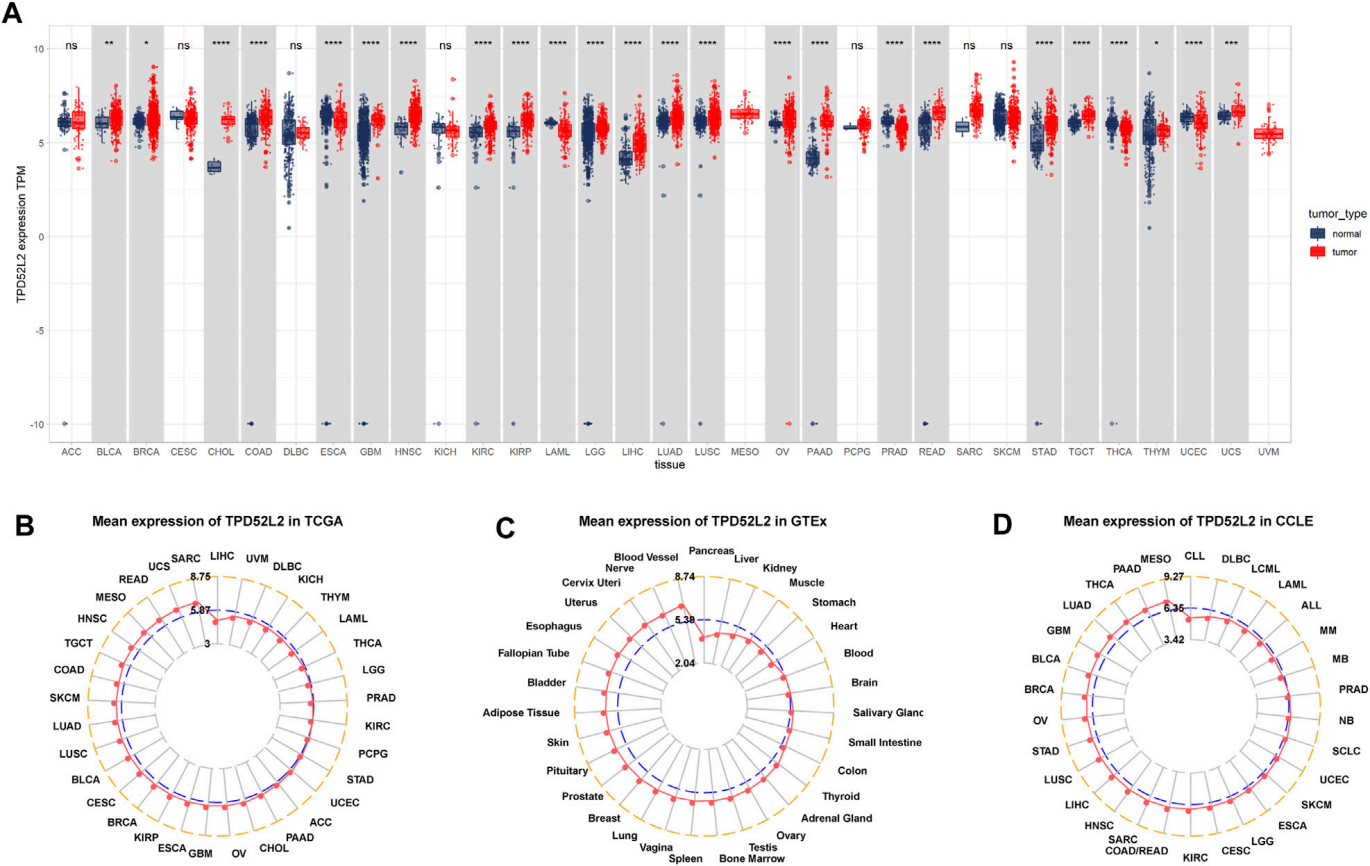
Expression of TPD52L2. **(A)** Pan-cancer expression of TPD52L2. **(B)** TPD52L2 expression in tumor tissues from TCGA cohort. **(C)** TPD52L2 expression in normal tissues from the GTEx cohort. **(D)** TPD52L2 expression in cancer cell lines from the CCLE cohort. *p < 0.05, **p < 0.01, ***p < 0.001, and ****p < 0.0001.

Firstly, we explored the expression of TPD52L2 in 33 tumor types using TCGA and GTEx data. The results revealed that TPD52L2 expression was significantly increased in 19 of 33 tumor types, including bladder urothelial carcinoma (BLCA), breast invasive carcinoma (BRCA), colon adenocarcinoma (COAD), cholangiocarcinoma (CHOL), glioblastoma multiforme (GBM), head-and-neck squamous cell carcinoma (HNSC), kidney renal clear cell carcinoma (KIRC), kidney renal papillary cell carcinoma (KIRP), brain lower grade glioma (LGG), liver hepatocellular carcinoma (LIHC), lung adenocarcinoma (LUAD), lung squamous cell carcinoma (LUSC), ovarian serous cystadenocarcinoma (OV), pancreatic adenocarcinoma (PAAD), rectum adenocarcinoma (READ), stomach adenocarcinoma (STAD), testicular germ cell tumor (TGCT), thymoma (THYM), and uterine carcinosarcoma (UCS), while it was only lowly expressed in esophageal carcinoma (ESCA), acute myeloid leukemia (LAML), pancreatic adenocarcinoma (PAAD), thyroid carcinoma (THCA), and uterine corpus endometrial carcinoma (UCEC) (Figure 1A). The difference analysis results are supplied in Supplementary Table S2. To assess TPD52L2 expression only in tumor tissues, we observed that TPD52L2 was highest in sarcoma (SARC) and lowest in LIHC (Figure 1B). In normal tissues from the GTEx database, the results revealed that TPD52L2 expression was highest in blood vessels and lowest in the pancreas (Figure 1C). As for tumor cell lines, we found that TPD52L2 expression was highest in mesothelioma (MESO) cell lines using data from the CCLE database (Figure 1D).

**FIGURE 2:**
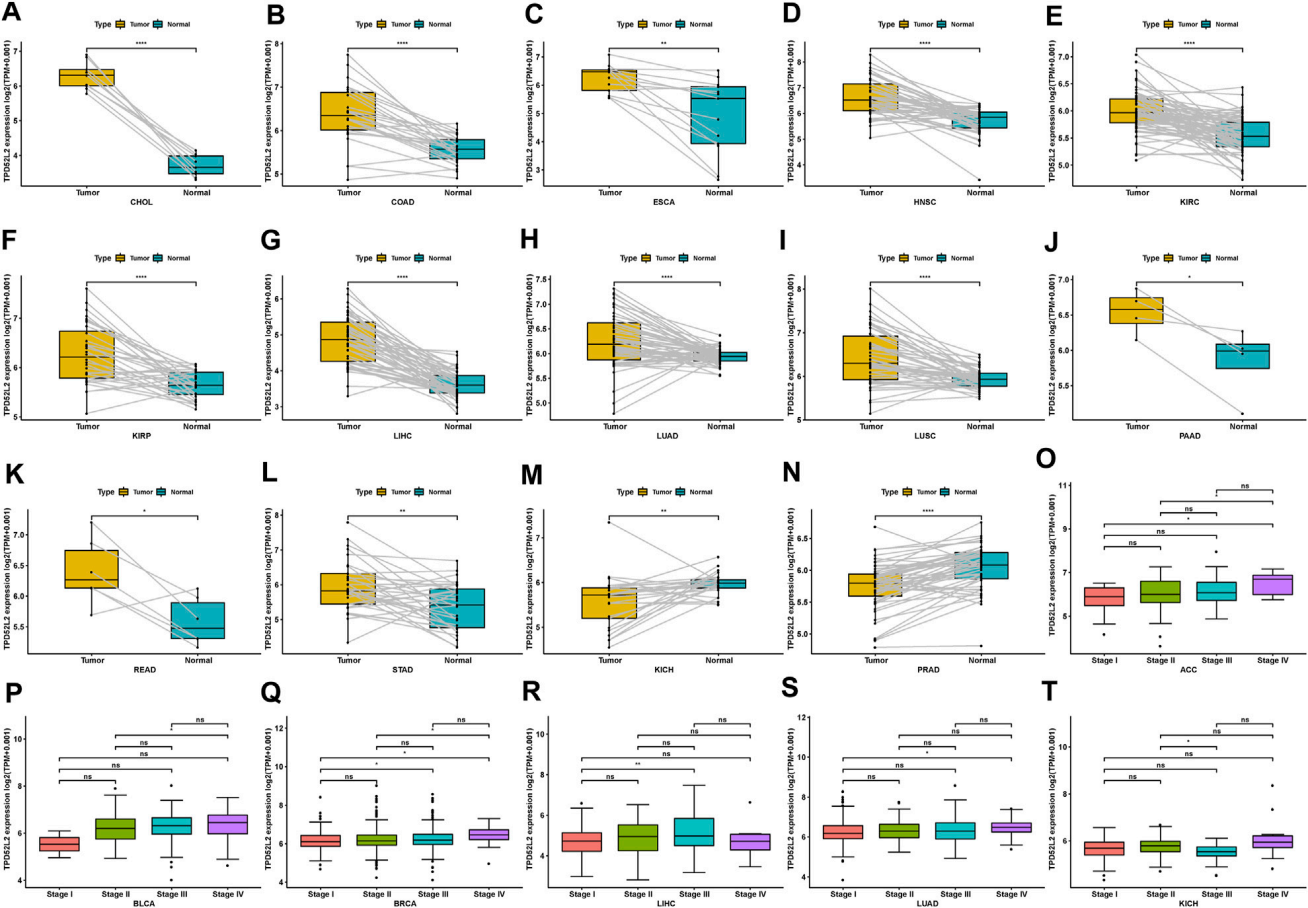
Expression of TPD52L2 in paired tumor and adjacent normal tissues. **(A–N)** TPD52L2 expression in paired tumor and adjacent normal tissues from TCGA in indicated tumor types. **(O–T)** TPD52L2 expression in various tumor stages in indicated tumor types. *p < 0.05, **p < 0.01, ***p < 0.001, and ****p < 0.0001.

We further evaluated TPD52L2 expression in paired tumor and normal tissues and various tumor stages. TPD52L2 expression was upregulated in tumor tissues in CHOL, COAD, ESCA, HNSC, KIRC, KIRP, LIHC, LUAD, LUSC, PAAD, READ, and STAD (Figures 2A–L). In contrast, TPD52L2 was lowly expressed in tumor tissues of KICH and PRAD (Figures 2M,N). In addition, TPD52L2 expression was higher in relatively worse tumor stages in adrenocortical carcinoma (ACC), BLCA, BRCA, LIHC, and LUAD, while it was lower in relatively worse tumor stages in KICH (Figures 2O–T).

### Gene Alteration of TPD52L2

**FIGURE 3:**
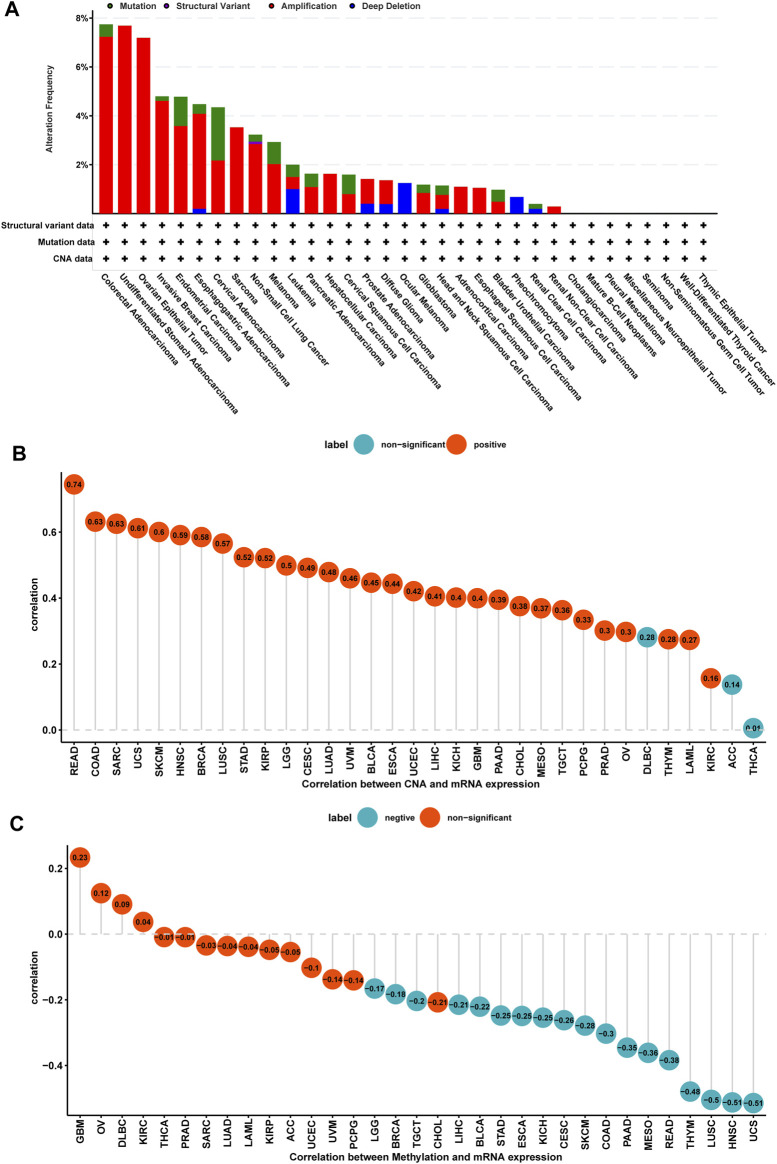
Gene alteration of TPD52L2. **(A)** Mutation and CNA status of TPD52L2 in TCGA-pan-cancer using the cBioPortal database. **(B)** Correlation between TPD52L2 expression and CNA. **(C)** Correlation between TPD52L2 expression and DNA methylation.

We further evaluated the mutation, CNA, and methylation status of TPD52L2 in pan-cancer. We found that the genomic alteration frequency of TPD52L2 was more than 2% in NSCLC patients, in which “amplification” was the primary type (Figure 3A). For the correlation between TPD52L2 and CNA, we found that TPD52L2 expression was positively correlated with CNA in LUAD (r = 0.48, *p* = 1.38E-30) (Figure 3B). We further proved that the promoter methylation level of TPD52L2 has little relationship with TPD52L2 expression (r = -0.04, *p* > 0.05) in LUAD (Figure 3C).

### Prognostic Significance of TPD52L2

**FIGURE 4:**
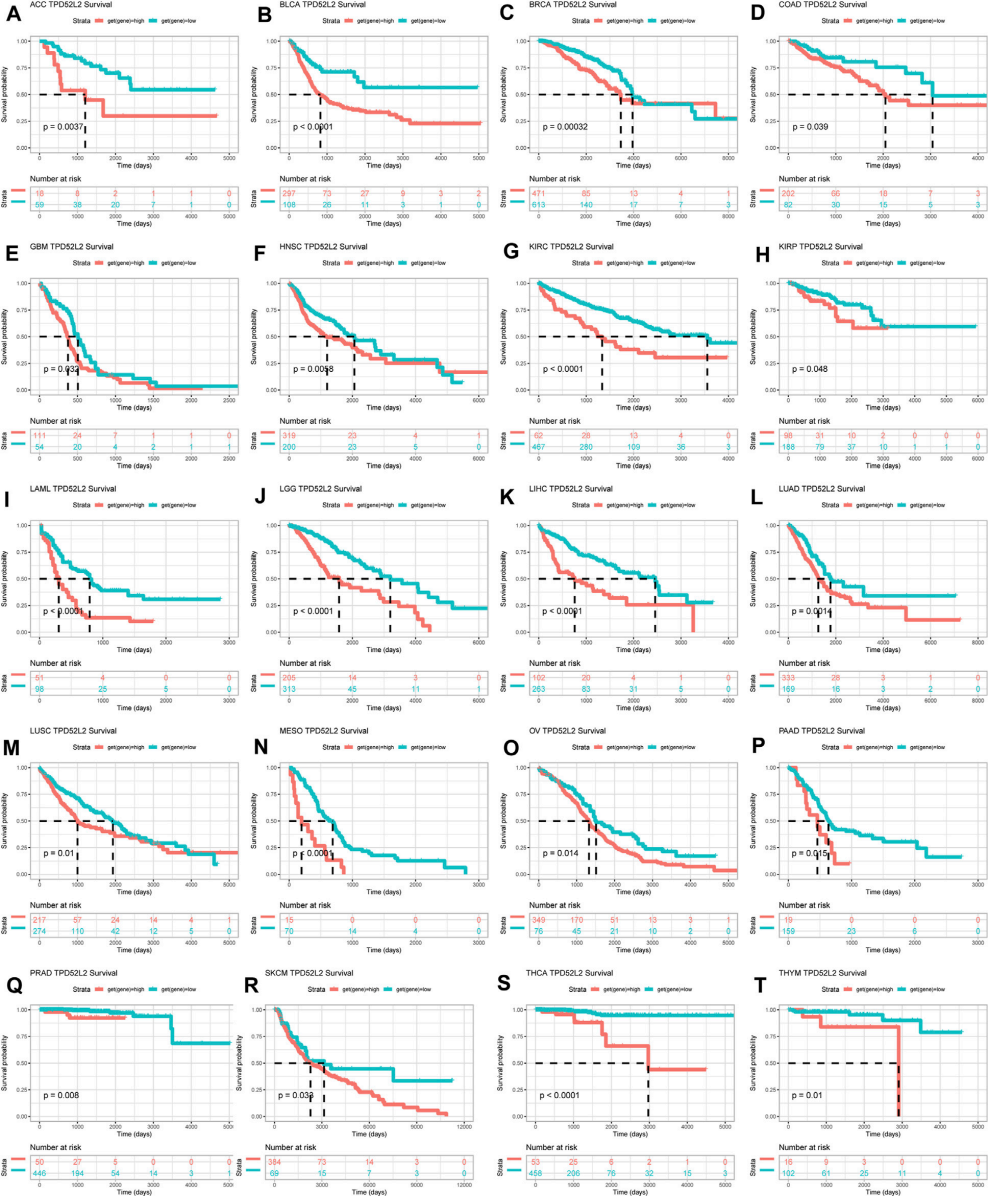
Prognostic significance of TPD52L2 for OS of patients. **(A–T)** Kaplan–Meier OS results of TPD52L2 in pan-cancer. The best cutoff of TPD52L2 expression was set as a cut-off value. Only significant results are shown.

To evaluate the prognostic significance of TPD52L2 in pan-cancer, we performed the Kaplan–Meier analysis and uniCox. For the Kaplan–Meier analysis of OS, we observed that the evaluated TPD52L2 expression predicted worse OS of patients in 20 of 33 tumors in TCGA cohort, including ACC, BLCA, BRCA, COAD, GBM, HNSC, KIRC, KIRP, LAML, LGG, LIHC, LUAD, LUSC, MESO, OV, PAAD, PRAD, SKCM, THCA, and THYM (Figure 4).

**FIGURE 5:**
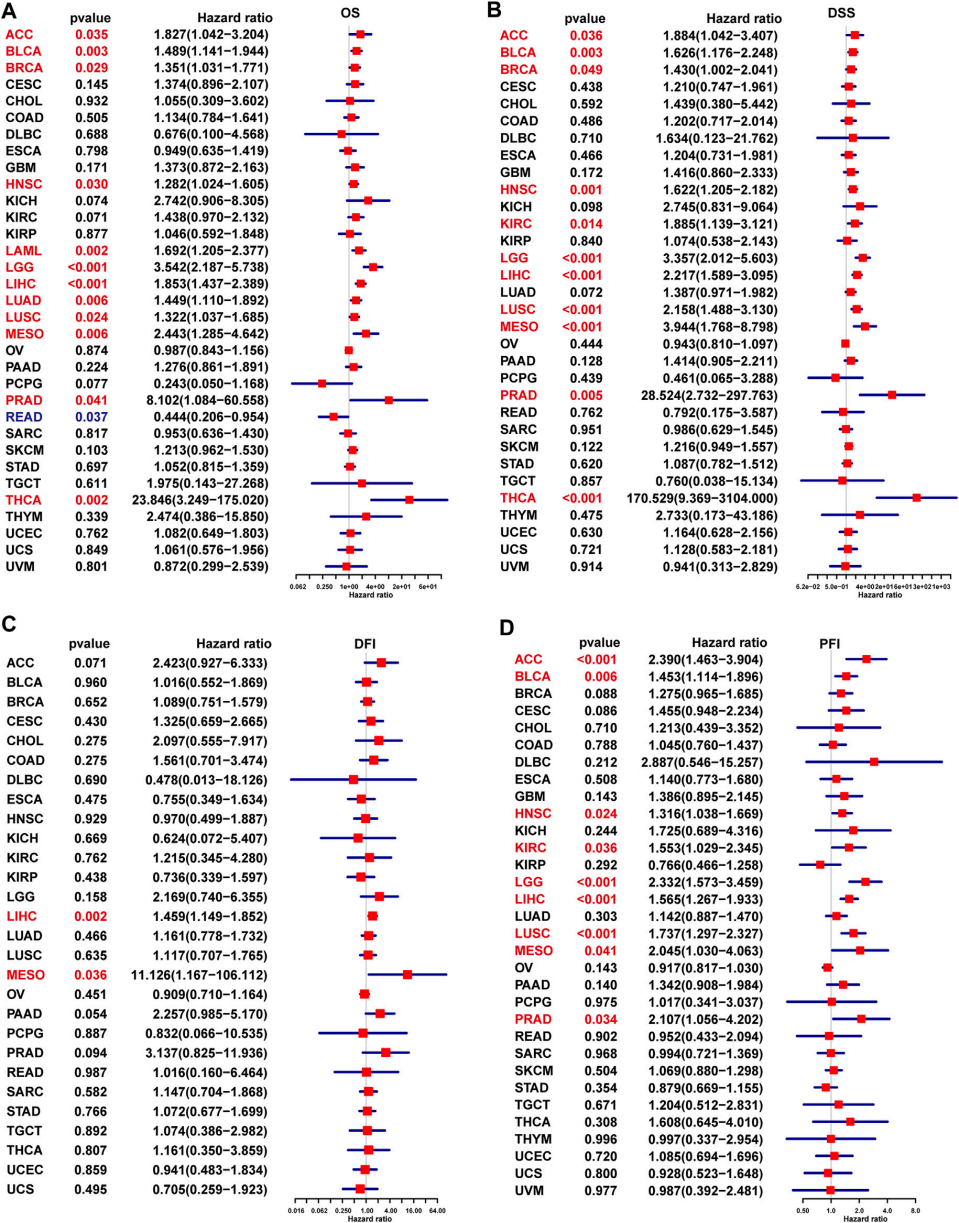
Prognostic significance of TPD52L2 for OS, DSS, DFI, and PFI of patients. **(A–D)** uniCox results of TPD52L2 in pan-cancer for OS **(A)**, DSS **(B)**, DFI **(C)**, and PFI **(D)** of patients. Red color represents significant results (*p* < 0.05).

In addition, the uniCox OS results indicated that TPD52L2 was a risk factor in ACC, BLCA, BRCA, HNSC, LAML, LGG, LIHC, LUAD, LUSC, MESO, PRAD, and THCA (Figure 5A). For DSS results, TPD52L2 was a risk factor in ACC, BLCA, BRCA, HNSC, KIRC, LGG, LIHC, LUSC, MESO, PRAD, and THCA (Figure 5B). For the DFI, high TPD52L2 expression predicted shorter DFI times in patients with LIHC and MESO (Figure 5C). For the PFI, high TPD52L2 expression predicted a worse PFI status in patients with ACC, BLCA, HNSC, KIRC, LGG, LIHC, LUSC, MESO, and PRAD (Figure 5D).

### GSEA of TPD52L2

**FIGURE 6:**
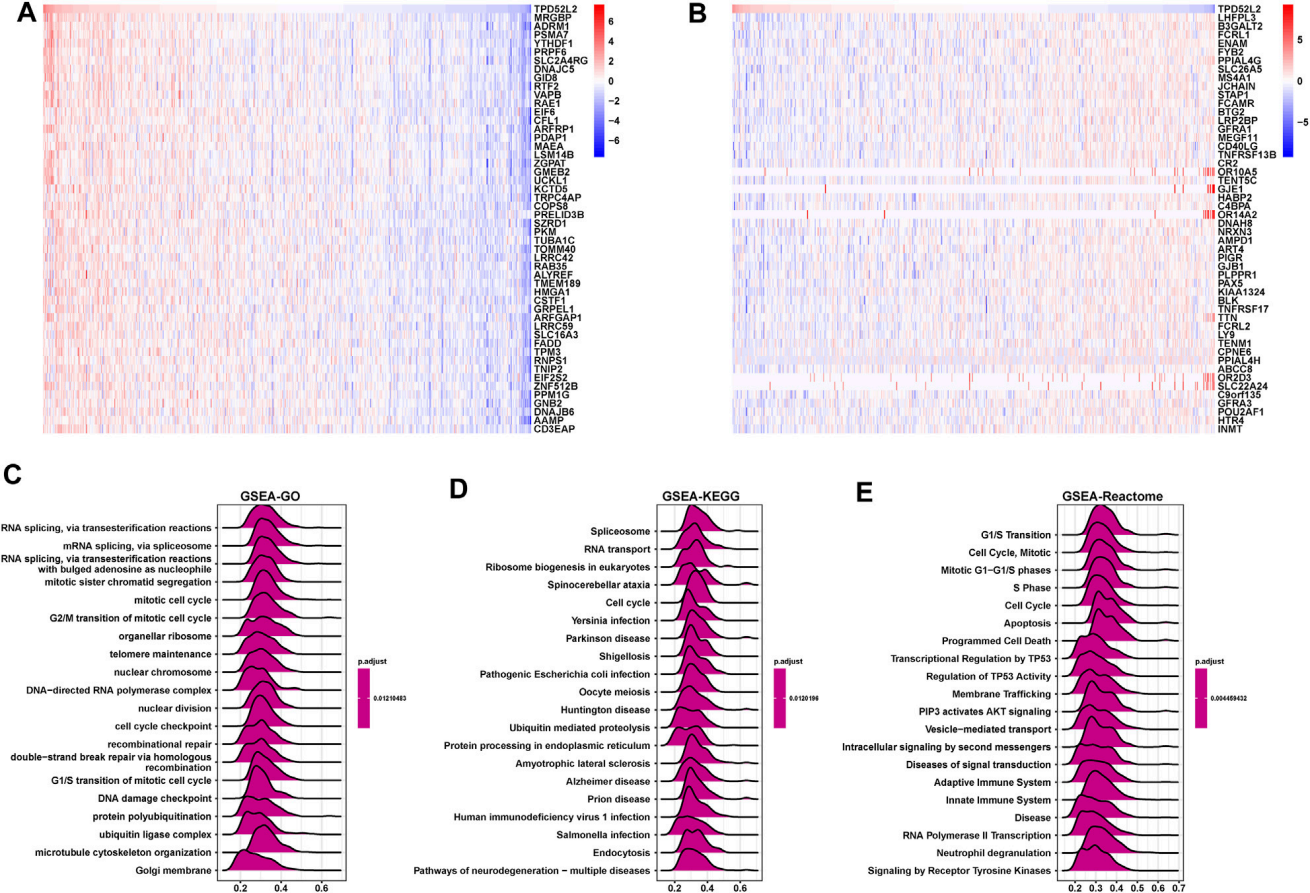
GSEA of TPD52L2. **(A)** Expression of top 50 genes positively correlated with TPD52L2 expression in LUAD. **(B)** Expression of top 50 genes negatively correlated with TPD52L2 expression in LUAD. **(C)** The top 20 GSEA-GO results are shown in LUAD. **(D)** The top 20 GSEA-KEGG results are shown in LUAD. **(E)** The top 20 GSEA-Reactome results are shown in LUAD.

Next, we conducted the GSEA to predict the pathways TPD52L2 might involve in. The genes correlated with TPD52L2 (*p* < 0.05) were ranked and used to perform GSEA (Figures 6A,B). We analyzed the Gene Ontology (GSEA-GO), Kyoto Encyclopedia of Genes and Genomes (GSEA-KEGG), and reactome pathway (GSEA-Reactome) terms using the R package “clusterProfiler” in LUAD (Figures 6C–E). The GSEA-GO results revealed that TPD52L2 was enriched in the function of “RNA splicing, *via* transesterification reactions” and “G2/M transition of mitotic cell cycle” terms. For GSEA-KEGG, TPD52L2 was associated with “Spliceosome” and “RNA transport” terms. For the results of GSEA-Reactome, we found that TPD52L2 was associated with cell cycle and immune-regulation–related pathways, such as “G1/S Transition,” “Cell Cycle,” “Adaptive Immune System,” and “Innate Immune System.”

### Immune Cell Infiltration Analysis of TPD52L2

**FIGURE 7:**
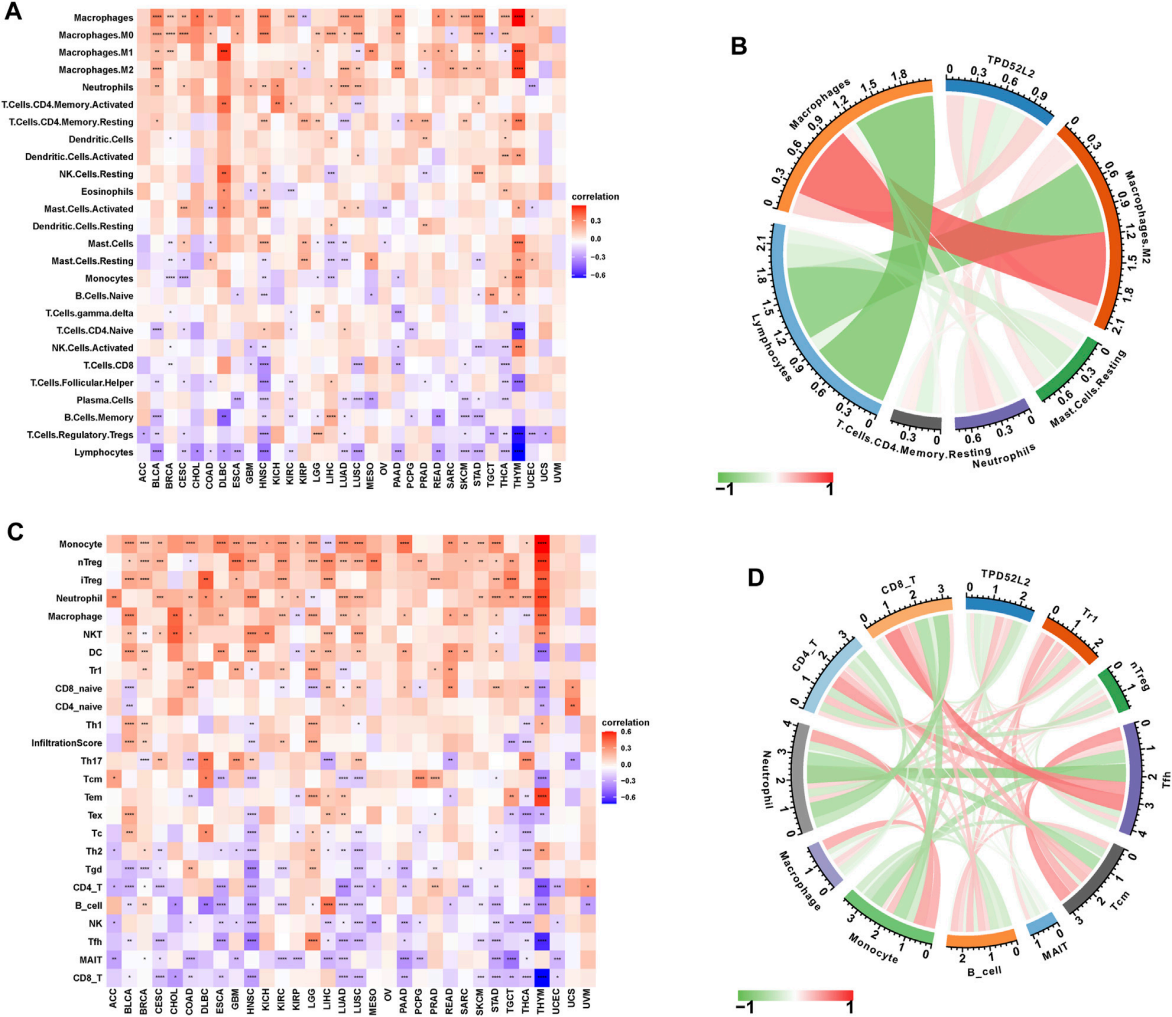
Immune infiltration analysis. **(A)** Correlation between TPD52L2 expression and infiltration levels of 26 immune cells downloaded from a published work in pan-cancer. Red represents positive correlation, blue represents negative correlation, and the darker the color, the stronger the correlation. **(B)** Correlation between TPD52L2 expression and indicated immune cells downloaded from a published work in LUAD. **(C)** Correlation between TPD52L2 expression and infiltration levels of 24 immune cells from ImmuCellAI in pan-cancer. **(D)** Correlation between TPD52L2 expression and indicated immune cells in the ImmuCellAI cohort in LUAD.

To prove the immune-regulation function of TPD52L2, we downloaded the infiltration level of 26 immune cells from the published research (Thorsson et al., 2018). The results of the correlation analysis suggested that TPD52L2 expression was positively correlated with TAMs and M2-like TAMs in LUAD (Figures 7A,B). We further obtained the 24 immune cells from the ImmuCellAI database. The results of the correlation analysis suggested that the expression level of TPD52L2 was positively correlated with immunosuppressive cells, such as Tregs and TAMs in LUAD. In contrast, TPD52L2 expression was negatively correlated with immune killer cells, including natural killer (NK) cells and CD8^+^ T cells in LUAD (Figures 7C,D).

**FIGURE 8:**
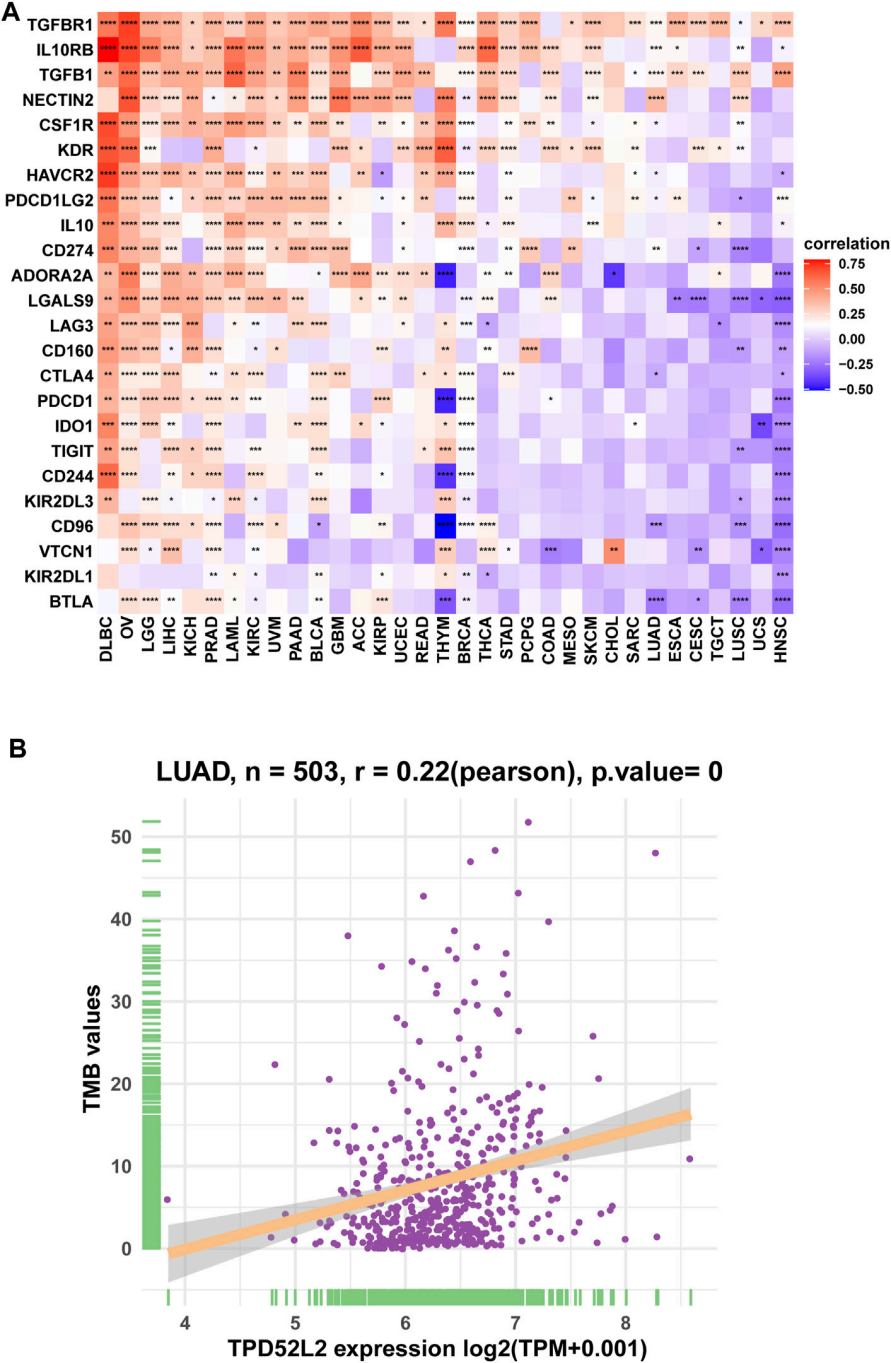
Correlation between immunosuppressive genes. **(A)** Correlation between TPD52L2 expression and immunosuppressive genes. **(B)** Correlation between TPD52L2 expression and TMB values. *p < 0.05, **p < 0.01, ***p < 0.001, and ****p < 0.0001.

We further proved that TPD52L2 expression was positively correlated with immunosuppressive genes in pan-cancer (Figure 8A), such as cluster of differentiation 274 (CD274), nectin cell adhesion molecule 2 (NECTIN2), transforming growth factor beta-1 (TGFB1), and transforming growth factor beta receptor-1 (TGFBR1). These results revealed that patients with high TPD52L2 expression might be in a relatively immunosuppressive environment.

### Correlation With Tumor Mutational Burden (TMB)

TMB values have essential connections with the sensitivity of ICIs. Therefore, we analyzed the correlation between TPD52L2 and TMB values and revealed that TPD52L2 was positively correlated with TMB values (Figure 8B).

### Gene Mutant Frequency in High- and Low-TPD52L2 Groups

**FIGURE 9:**
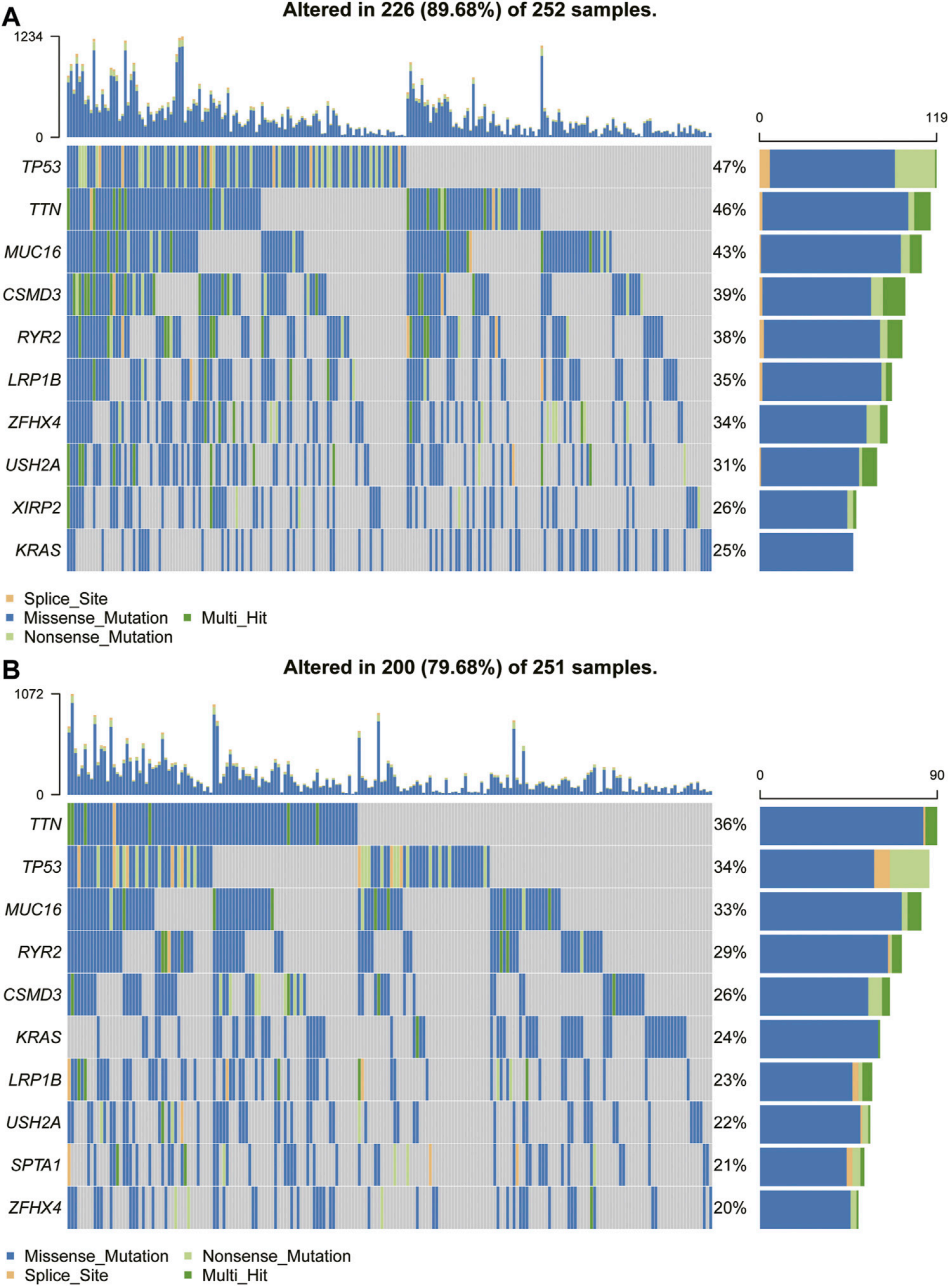
Gene mutant frequency in high- and low-TPD52L2 groups. **(A)** Gene mutant frequency (top 10) in the high TPD52L2 expression group. **(B)** Gene mutant frequency (top 10) in the low TPD52L2 expression group.

Because the prognosis of patients with high expression of TPD52L2 is worse than that of patients with low expression of TPD52L2, we speculated whether the expression of TPD52L2 is related to the mutations of some common cancer-promoting genes, so we further analyzed the gene mutations (such as TP53, MUC16, and TTN) in high and low TPD52L2 expression groups (Figures 9A,B). We proved that the expression of TPD52L2 was potentially related to the mutation of TP53, MUC16, and TTN genes. For example, there are 252 samples and 251 in high and low TPD52L2 expression groups (high and low expression groups according to the median score of TPD52L2 expression), of which 119 and 86 samples have TP53 mutation, so the mutation frequency of TP53 in TPD52L2 high and low expression groups is 47 and 34%. The TP53 mutation frequency was higher in the high TPD52L2 expression group (*p* = 0.0037, Supplementary Table S3).

## Discussion

The tumor protein D52 (TPD52) family has important biological functions in several cancers (Balleine et al., 2000; Wang et al., 2016). TPD52L2, also known as TPD54 and D54, is a member of the TPD52 family (Boutros et al., 2004). Recent research shows that the expression levels of TPD52L2 were upregulated in prostate cancer tissues, andincreased TPD52L2 expression is associated with clinical progression and poor prognosis in patients with prostate cancer, suggesting that TPD52L2 may be a potential prognostic marker for patients with prostate cancer (Ren et al., 2017). A previous study demonstrated that knockout of TPD52L2 inhibits glioma cell proliferation by arresting cells in the G0/G1 phase (Wang et al., 2014). However, the expression pattern and biological role of TPD52L2 in LUAD have not been studied until now.

In our study, we firstly assessed the expression of TPD52L2 and found TPD52L2 expression was higher in tumor tissues compared with normal tissues in LUAD. In addition, TPD52L2 expression was higher in relatively worse tumor stages in LUAD. Meanwhile, the “amplification” of TPD52L2 CNA occurs most frequently in LUAD. Next, we found that TPD52L2 expression was positively correlated with CNA in LUAD, indicating that the CNA status mainly contributes to the high expression of TPD52L2. An elevated TPD52L2 expression predicted poorer OS of patients with LUAD. These results suggest that TPD52L2 may serve an important role in the onset and development of LUAD. We performed the GSEA and found that TPD52L2 was associated with cell cycle and immune-regulation–related pathways, such as “G1/S Transition,” “Cell Cycle,” “Adaptive Immune System,” and “Innate Immune System.” These results indicated that cell cycle and immune-regulation function of TPD52L2 may induce the poorer survival status of patients with LUAD.

Furthermore, we further explored the potential role of TPD52L2 in regulating the TME. Increasing evidence has revealed that the level of immune cell infiltration in the TME is a critical determinant in tumor development and therapeutic response of cancer (Suzuki et al., 2021). These infiltrating immune cells, such as tumor-associated macrophages (TAMs) and regulatory T cells (Tregs), could promote cancer progression and immune escape by secreting several inflammatory cytokines and chemokines (Cully, 2018). Thus, we further performed a correlation analysis between immune cell infiltration levels and TPD52L2 expression. The results suggested that TPD52L2 was positively correlated with TAM and Treg infiltration, indicating that TPD52L2 influences the prognosis of patients with LUAD probably *via* its interaction with infiltrating immune cells. TGFB1 has been proved as the most predominant immune-suppressing molecule in the TME. TGFB1 can inhibit the generation, differentiation, and function of effector T cells, as well as inducing Treg infiltration into the TME (Woo et al., 2015). CD274, also called programmed cell death ligand-1 (PD-L1), has been widely demonstrated to inhibit anti-tumor immunity (Johnson et al., 2017). Our results showed that TPD52L2 expression was positively correlated with immunosuppressive genes in LUAD, such as CD274, TGFB1, and TGFBR1. These results revealed that high TPD52L2 expression is a possible indicator of immune infiltration and associated with tumor immunosuppressive status in LUAD.

Recent years, TMB has been discovered as a promising biomarker in a variety of tumor immunotherapies and can be used to predict the efficacy of immunotherapy (Devarakonda et al., 2018; Choucair et al., 2020). Those with high TMB expression have been shown to benefit more from immune checkpoint inhibitor therapy (Yarchoan et al., 2017). In addition, we analyzed the correlation between TPD52L2 and TMB values and revealed that TPD52L2 was positively correlated with TMB values. TP53 mutations frequently occur in cancer and are associated with poor prognosis in a wide variety of cancers (Dong et al., 2017). In lung cancer immunotherapy, TP53 mutation can be recognized as a predictor of immunotherapy sensitivity (Skoulidis et al., 2018). We also proved that LUAD patients with high expression of TPD52L2 were with more TP53 mutation rates, suggesting that patients with high TPD52L2 expression may be a suitable biomarker for immunotherapy in LUAD.

In conclusion, our findings revealed that TPD52L2 was upregulated and could act as a prognostic marker in LUAD. High TPD52L2 expression was associated with the infiltration levels of immunosuppressive cells and immunosuppressive genes. These findings demonstrated there was great potential for TPD52L2 acting as a biomarker for prognosis and a target for tumor therapy in LUAD.

## Data Availability

The original contributions presented in the study are included in the article/Supplementary Material, and further inquiries can be directed to the corresponding author.
